# Dose Reduction in Scintigraphic Imaging Through Enhanced Convolutional Autoencoder-Based Denoising

**DOI:** 10.3390/jimaging11060197

**Published:** 2025-06-14

**Authors:** Nikolaos Bouzianis, Ioannis Stathopoulos, Pipitsa Valsamaki, Efthymia Rapti, Ekaterini Trikopani, Vasiliki Apostolidou, Athanasia Kotini, Athanasios Zissimopoulos, Adam Adamopoulos, Efstratios Karavasilis

**Affiliations:** 1 Medical Physics Laboratory, School of Medicine, Democritus University of Thrace, 69100 Alexandroupolis, Greece; katetriko@gmail.com (E.T.); akotini@med.duth.gr (A.K.); adam@med.duth.gr (A.A.); 2 Nuclear Medicine Department, University General Hospital of Alexandroupolis, Dragana, 69100 Alexandroupolis, Greece; pivalsam@med.duth.gr (P.V.); raptiefthymia@gmail.com (E.R.); bapostolid@gmail.com (V.A.); 3 2nd Department of Radiology, Medical School, Attikon University Hospital, National and Kapodistrian University of Athens, 11527 Athens, Greece; ianstath@med.uoa.gr; 4 Nuclear Medicine Department, Medical School, Democritus University of Thrace, Dragana, 69100 Alexandroupolis, Greece; azisimop@med.duth.gr

**Keywords:** bone scintigraphy, convolutional autoencoder, deep learning, image denoising, low-dose imaging, nuclear medicine, artificial intelligence

## Abstract

Objective: This study proposes a novel deep learning approach for enhancing low-dose bone scintigraphy images using an Enhanced Convolutional Autoencoder (ECAE), aiming to reduce patient radiation exposure while preserving diagnostic quality, as assessed by both expert-based quantitative image metrics and qualitative evaluation. Methods: A supervised learning framework was developed using real-world paired low- and full-dose images from 105 patients. Data were acquired using standard clinical gamma cameras at the Nuclear Medicine Department of the University General Hospital of Alexandroupolis. The ECAE architecture integrates multiscale feature extraction, channel attention mechanisms, and efficient residual blocks to reconstruct high-quality images from low-dose inputs. The model was trained and validated using quantitative metrics—Peak Signal-to-Noise Ratio (PSNR) and Structural Similarity Index (SSIM)—alongside qualitative assessments by nuclear medicine experts. Results: The model achieved significant improvements in both PSNR and SSIM across all tested dose levels, particularly between 30% and 70% of the full dose. Expert evaluation confirmed enhanced visibility of anatomical structures, noise reduction, and preservation of diagnostic detail in denoised images. In blinded evaluations, denoised images were preferred over the original full-dose scans in 66% of all cases, and in 61% of cases within the 30–70% dose range. Conclusion: The proposed ECAE model effectively reconstructs high-quality bone scintigraphy images from substantially reduced-dose acquisitions. This approach supports dose reduction in nuclear medicine imaging while maintaining—or even enhancing—diagnostic confidence, offering practical benefits in patient safety, workflow efficiency, and environmental impact.

## 1. Introduction

Modalities for nuclear medicine imaging techniques, including gamma cameras, technologically advanced Anger camera-based Single Photon Emission Computed Tomography (SPECT), and Positron Emission Tomography (PET), play a crucial role in assessing disease appearance or progression, lesion malignancy, and other biochemical or metabolic processes within the human organism [1]. These modalities utilize radiopharmaceuticals—pharmaceutical substances labeled with radioactive isotopes—to provide functional insights even before structural alterations occur. Bone scintigraphy is one of the most widely used nuclear medicine techniques, particularly for detecting bone metastases, fractures, infections, metabolic bone disease, primary bone tumors, and other skeletal abnormalities [2]. One of the primary goals in nuclear medicine is to minimize radiation exposure to both patients and healthcare professionals. To achieve this objective, efforts are continually made to reduce the administered radiopharmaceutical dose. However, reducing the dose often leads to image noise due to undersampling, which can compromise diagnostic accuracy [3,4]. Given that bone scintigraphy is extensively used in oncology and orthopedics, enhancing the quality of low-dose bone scintigraphy images is of significant clinical importance, leading simultaneously to reduced patients’ and employees’ radiation burden and to increased diagnostic accuracy [5]. Technological advancements, particularly in artificial intelligence (AI), offer promising solutions to address these challenges. AI-based techniques are increasingly employed to enhance image reconstruction, reduce noise, and improve overall diagnostic reliability, even in low-dose imaging in radiology and nuclear medicine [4,6,7,8,9]. To the best of our knowledge, although numerous algorithms have been developed for radiology, commercial and non-commercial AI tools specifically tailored for nuclear medicine remain limited. The scientific community has primarily used convolutional neural networks (CNNs) and other deep learning models, such as Conditional and General Generative Adversarial Networks (GANs), to denoise bone and heart scintigraphy, as well as PET images, achieving high efficiency [6,8,9,10,11,12,13]. Furthermore, Convolutional Autoencoders (Conv-Autoencoders), which are often designed for unsupervised learning tasks, have gained attention in recent studies due to their ability to learn compact representations of noisy images and perform effective denoising, further enhancing the quality of medical images. These models capture spatial hierarchies and allow for effective noise reduction while preserving critical structural information, making them valuable for applications in low-dose imaging [14,15]. However, recent work using Conditional Generative Adversarial Networks (cGANs) for denoising medical images has also shown strong results, relying on supervised learning to improve image quality in a controlled setting [13]. Scintigraphically noisy images, in which algorithms have been trained and validated, were ordinarily based on simulated anthropomorphic, phantom images, and rarely real imaging data, resulting in degraded high-quality images either from sub-sampling acquisition protocols or reduced administered radiopharmaceutical doses [5,6,7,11,12,13].

Thus, the aim of this study was to develop an AI-driven technique, trained and validated on real-world images, to reduce the administered dose and patients’ radiation burden in nuclear medicine scintigraphy applications.

## 2. Materials and Methods

### 2.1. Ethics and Compliance

This study was conducted in full compliance with the General Data Protection Regulation (GDPR) [EU 2016/679] of 25 May 2018, regarding the protection of sensitive personal data. Prior to its implementation, the necessary approvals were obtained from the relevant authorities. The data collected were fully anonymized and used exclusively for research purposes, with access restricted to the principal investigator. Participants provided written informed consent after being fully informed that the process was anonymous, their personal information and responses would be used solely for research purposes, and that they could withdraw from the study at any time [16].

### 2.2. Dataset

The data collection was conducted at the Nuclear Medicine Department of the University General Hospital of Alexandroupolis in 2023. This study utilizes a dataset of static images from bone scintigraphy. Anonymous images were collected from 105 bone scintiscans (44 males and 61 females). The age range of the examined individuals was 37 to 88 years, with a mean age of 67 ± 11.29 years. The mean patient weight was 74.78 ± 12.11 kg, ranging from 53 kg to 106 kg, reflecting a typical adult population in clinical nuclear medicine practice.

The data were acquired using two gamma cameras: a Siemens Symbia E Dual Head System (Siemens Healthineers, Erlangen, Germany) and a Siemens E-CAM e-signature (Siemens Healthineers, Erlangen, Germany). The imaging parameters and procedures followed were in accordance with standard clinical practice. Patients were intravenously injected with hydroxyethylene diphosphonate (HDP) labeled with the metastable radioisotope technetium 99m (^99m^Tc). The administered activity ranged from 630 to 740 MBq (17–20 mCi), depending on patient body weight. Static images were acquired with a matrix size of 256 × 256, a zoom factor of 1, and a 15% energy window centered at the 140 keV photopeak. To minimize the influence of dose variability on model input, all images were intensity normalized during preprocessing in a standard manner. As such, differences in administered activity had negligible impact on the training or evaluation process. To promote clinical practicability and generalizability, no images were excluded based on patient weight, pathology, or presence of medical devices such as urinary catheters. The dataset thus reflects a heterogeneous clinical population, including both abnormal and healthy cases. While extreme or rare cases were limited, this approach aims to develop a denoising model applicable across typical real-world scenarios. In this context, extreme or rare cases refer to patients with substantial anatomical abnormalities (e.g., major limb amputations) or significant skeletal deformities. The two gamma cameras differ slightly in design and acquisition characteristics, with the newer Siemens Symbia E (Siemens Healthineers, Erlangen, Germany) generally expected to produce images with marginally higher baseline quality. To assess any impact of scanner differences on denoising performance, image quality metrics including SSIM and PSNR were compared across the two devices, revealing comparable results (Appendix A Table A1). This suggests the model generalizes well across these scanner models within our dataset.

### 2.3. Acquisition Protocol

**Figure 1:**
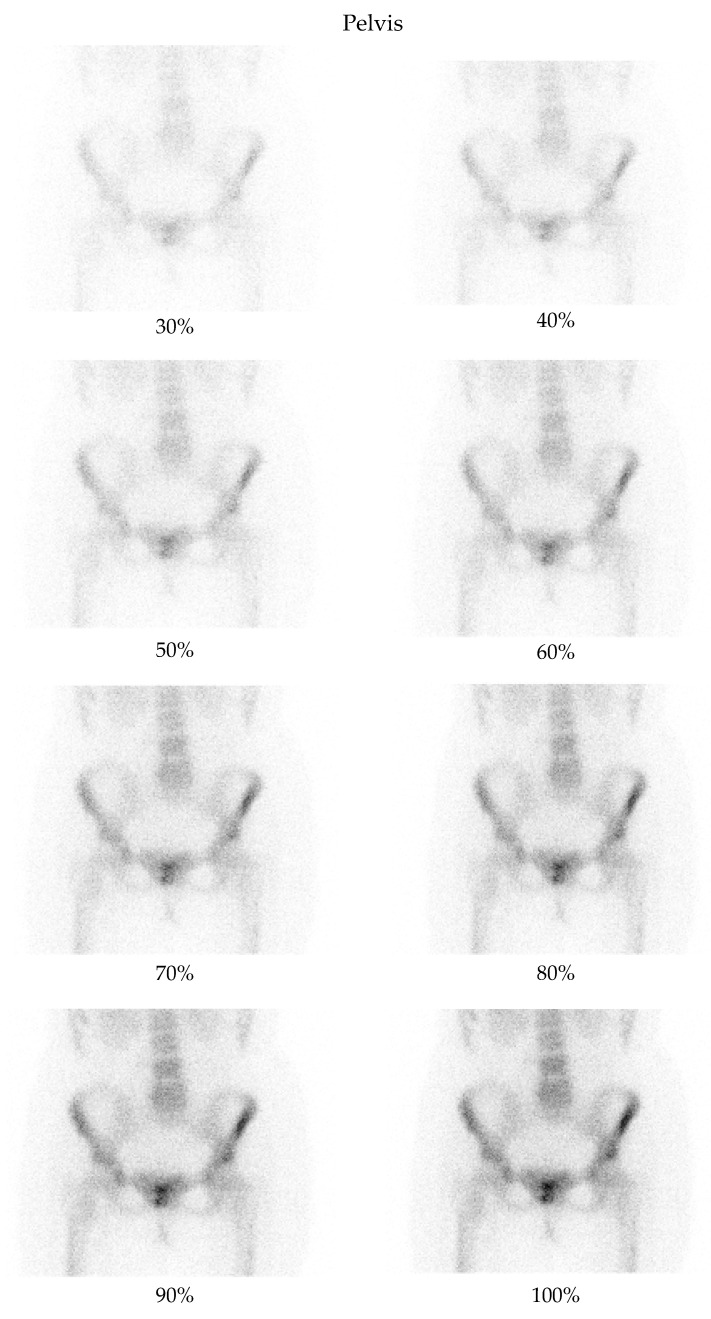
Bone scintigraphy planar images of the pelvis, showing an example of degraded low-dose images as compared with the corresponding full-dose image.

**Figure 2:**
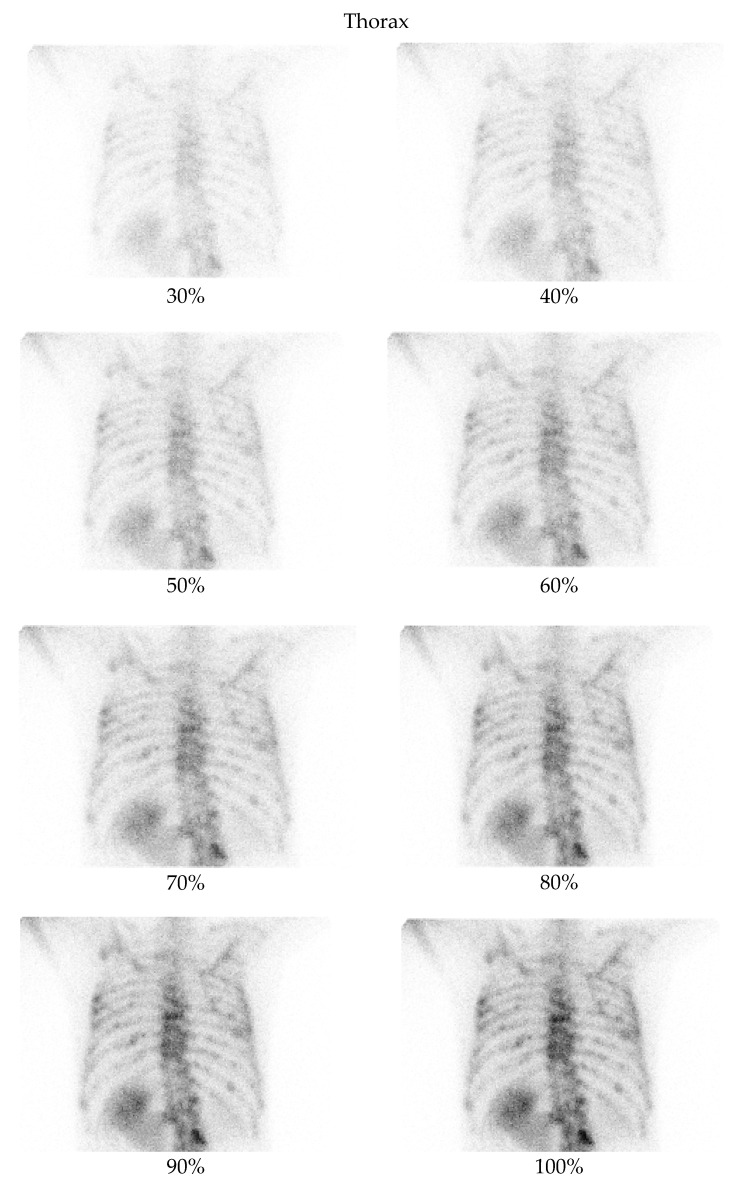
Bone scintigraphy planar images of the thorax, showing an example of degraded low-dose images as compared with the corresponding full-dose image.

A low-dose imaging protocol was developed using parameters identical to the clinical protocol in routine nuclear medicine practice, with the exception that images were captured at 30%, 40%, 50%, 60%, 70%, 80%, 90%, and 100% of the standard acquisition time. This reduction in acquisition time simulates a corresponding percentage reduction in dose. The hypothesis was confirmed experimentally using a point source with an activity of 2.24 mCi. Imaging was performed according to the established protocol, capturing images at each of these acquisition times and comparing the number of counts in the images with the corresponding activity percentage. The results confirmed the correlation between acquisition time and dose reduction. Although this method does not replicate all clinical complexities (e.g., biological uptake, attenuation), it provides a practical physical basis for using time reduction as a proxy for dose reduction. The protocol was used to acquire images from two anatomical regions: the pelvis and the thorax (Figure 1 and Figure 2). For each region, two images (anterior and posterior) were simultaneously captured using the dual-head configuration of the gamma cameras.

#### Input–Output Pairing for Supervised Learning

This study employs a supervised learning approach in which paired images of the same anatomical region, captured at different acquisition times, are used to train the model. Specifically, each input image is a low-dose bone scintigraphy scan acquired at a reduced acquisition time (ranging from 30% to 90% of the standard duration), while the corresponding target image represents the full-dose scan (100% acquisition time) of the same view. These paired images simulate dose reduction through controlled acquisition protocols and provide the network with explicit input–output examples to learn the mapping from low-quality to high-quality images. This formulation justifies the supervised learning paradigm, as the model is trained to minimize the discrepancy between its output and the known high-quality ground truth using a predefined loss function. To ensure consistency in training data, input–output pairs were obtained from the same patient, anatomical region, and imaging session, using identical positioning protocols. The only controlled variation between paired images was our protocol-defined acquisition time, which was systematically reduced to simulate different dose levels. This design minimizes variability related to anatomical differences and imaging conditions. While no explicit stratification by pathology or clinical indication was performed, the dataset intentionally included a diverse range of skeletal abnormalities to promote model generalization. Minor inconsistencies due to patient motion or tracer kinetics may still occur and are recognized as a potential source of variability in training.

### 2.4. Enhanced Convolutional Autoencoder Model

#### 2.4.1. Model Architecture

**Figure 3:**
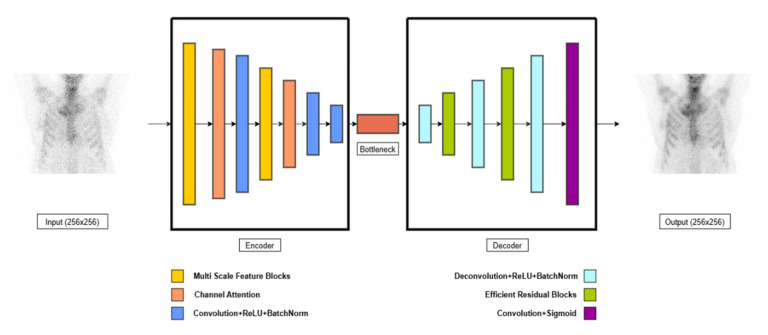
Schematic illustration of Enhanced Convolutional Autoencoder.

Convolutional Autoencoders are widely used in denoising applications for medical imaging due to their ability to learn efficient representations of image structure and suppress noise while preserving anatomical content [12,15]. The proposed Enhanced Convolutional Autoencoder (ECAE) (Figure 3) is designed to extract relevant features and reconstruct high-quality bone scintigraphy images from low-dose inputs [17]. The encoder consists of multi-scale feature extraction blocks (MSFBs), which apply parallel convolutional layers with varying kernel sizes (3 × 3, 5 × 5) to capture both fine-grained and contextual features [18]. Larger kernel sizes (e.g., 7 × 7 or 9 × 9) were not used to avoid excessive smoothing of fine structures and to maintain computational efficiency [19]. A channel attention mechanism is integrated after each MSFB, dynamically recalibrating feature importance through global average pooling (GAP) and fully connected layers with sigmoid activation [20] allowing the model to emphasize diagnostically relevant features. Instead of using traditional max pooling, strided convolutions are employed for downsampling. This choice preserves learnable spatial transformations and helps reduce information loss during compression, which is particularly important in medical imaging where anatomical fidelity is critical [21]. A 1 × 1 convolutional bottleneck compresses the latent feature map and facilitates efficient feature blending across channels; this is followed by a ReLU activation to introduce non-linearity and preserve learning capacity in the latent space. The decoder reconstructs the high-quality image using transposed convolutions. To prevent degradation of information across deeper layers, we incorporate efficient residual blocks (ERBs) within the decoder. These are termed “efficient” because they use depthwise separable convolutions and skip connections, reducing the number of trainable parameters while maintaining high expressiveness and stability during training. ERBs improve feature propagation and minimize gradient vanishing, leading to sharper reconstructions [22,23]. The final output is generated through a 3 × 3 convolution that produces the denoised grayscale image. The ECAE architecture was selected over more complex alternatives such as U-Nets, GANs, or transformer-based models due to its favorable balance between structural preservation, computational simplicity, and training stability. GANs are known for generating high-quality images but are more prone to training instability and hallucination effects, which can be problematic in clinical imaging [24,25]. U-Nets and transformers, while powerful, introduce significant parameter overhead and may be unnecessarily complex for the specific task of denoising [26,27]. In contrast, the ECAE offers interpretable feature representations and has proven to be efficient and robust under limited-data conditions, making it well suited for this clinical application [28]. This architectural design—combining multi-scale feature extraction, channel attention, residual learning, and bottleneck compression—allows the model to reconstruct diagnostically useful images with enhanced structural integrity and reduced noise, even at significantly reduced dose levels.

#### 2.4.2. Loss Function and Optimization

The model is trained using a hybrid loss function that combines Mean Squared Error (MSE) loss and Structural Similarity Index (SSIM) loss to balance pixel-wise accuracy and perceptual quality [29,30]. The total loss function is defined as

(1) $$L_{total}=\lambda \cdot L_{\mathrm{MSE}}+\left(1-\lambda \right)\cdot \left(1-\mathrm{SSIM}\left(X_{output},X_{target}\right)\right)$$

where

- *L*_MSE_ is the Mean Squared Error between predicted and target images,
- SSIM (*X_output_*, *X_target_*) is the Structural Similarity Index between the reconstructed image and the ground truth,
- *λ* is the weighting factor (default: 0.7), controlling the trade-off between pixel-wise fidelity and perceptual similarity,
- *X_output_* is the reconstructed (denoised) image,
- *X_target_* is the full-dose reference image.

The MSE ensures pixel-wise accuracy, while SSIM enhances structural preservation. The model is trained using the Adam optimizer [31,32] with a learning rate of 1 × 10^−3^ [33]. To avoid overfitting, early stopping is applied with a patience of 50 validation steps (i.e., iterations over validation batches)—training halts if no improvement is seen in validation loss across 50 consecutive validation checks. Additionally, input images are normalized to the range [0, 1] to ensure uniformity and improve the model’s stability during training.

#### 2.4.3. Training Setup

The proposed Enhanced Convolutional Autoencoder (ECAE) was implemented in PyTorch 2.6.0 [34] and trained on a Google Colab instance with an NVIDIA Tesla T4 GPU (NVIDIA Corporation, Santa Clara, CA, USA) [35]. The dataset used for training consists of grayscale images from 105 patients, each with a resolution of 256 × 256. These images include static bone scintigraphy scans from two anatomical regions (thorax and pelvis) and two views (anterior and posterior) for each region. For each region, images are captured at 8 different acquisition percentages (30%, 40%, 50%, 60%, 70%, 80%, 90%, and 100%), leading to a total of 16 images per region. The total number of paired images used is 1470 per anatomical region. The dataset is divided into training (75%), validation (15%), and test (10%) sets. The input images are normalized to the range [0, 1] before being fed into the network. The images are paired as input–output examples, with low-dose images (30–90%) as inputs and the corresponding full-dose (100%) images as the targets, as detailed in the Input–Output Pairing for Supervised Learning section. The training is conducted with a batch size of 16, and early stopping was applied based on validation loss, with a patience of 50 training iterations (i.e., mini-batch updates). Specifically, training was halted if the validation loss did not improve after 50 consecutive training steps during which validation was evaluated. The model was trained with a maximum of 2000 iterations with an initial learning rate of 1 × 10^−3^. Performance is evaluated using metrics such as Peak Signal-to-Noise Ratio (PSNR) and Structural Similarity Index (SSIM). A small number of image pairs were dropped during training due to compatibility constraints with the batch size and model input dimensions. These samples were randomly excluded to ensure that the dataset size was divisible by the batch size and to prevent tensor shape mismatches. Although data augmentation techniques (e.g., flipping, rotation, noise injection) are commonly employed to enhance dataset diversity, they were not used in this study. This choice was deliberate, given the clinical need to preserve anatomical realism in bone scintigraphy; artificial transformations could introduce unrealistic or misleading features. To further reduce the risk of overfitting, the model incorporated Group Normalization layers and residual connections to enhance generalization and training stability. While k-fold cross-validation was not employed, due to the substantial logistical demands of repeated expert scoring (including 2AFC evaluations and qualitative assessments), this represents a valuable direction for future studies, particularly with larger and more diverse datasets.

### 2.5. Performance Evaluation: Quantitative and Qualitative Metrics

The performance of the proposed model was quantitatively evaluated using two common image quality metrics: Peak Signal-to-Noise Ratio (PSNR) and Structural Similarity Index (SSIM).

1. PSNR (Peak Signal-to-Noise Ratio): PSNR is a measure of the quality of reconstruction, specifically assessing the pixel-wise accuracy between the reconstructed and original images. Higher PSNR values indicate a closer match between the images. The PSNR is defined as

(2) $$\mathrm{PSNR}=10\cdot \log_{10}\cdot \left(\frac{{I_{\mathrm{MAX}}}^{2}}{\mathrm{MSE}}\right)$$

where

- *I*_max_ is the maximum pixel value in the image (255 for 8-bit grayscale images),
- MSE is the Mean Squared Error (MSE) between the reconstructed and reference images,

MSE defined as

(3) $$\mathrm{MSE}=\frac{1}{N}\sum_{i=1}^{N}{\left(X_{output,i}-X_{target,i}\right)}^{2}$$

where

- *N* is the total number of pixels in the image,
- *X_output_* is the reconstructed (denoised) image,
- *X_target_* is the full-dose reference image.

A lower MSE value indicates better reconstruction accuracy [15,36].

2. SSIM (Structural Similarity Index): SSIM evaluates the perceived quality of the images by considering structural changes, luminance, and contrast. It is more perceptually meaningful than traditional metrics like MSE. SSIM ranges from −1 to 1, with a value closer to 1 indicating high structural similarity between the original and reconstructed images. The SSIM is defined as

(4) $$\mathrm{SSIM}\left(X_{output},X_{target}\right)=\frac{\left(2\mu_{Χ}\mu_{Y}+C_{1}\right)\left(2\sigma_{XY}+C_{2}\right)}{\left(\mu_{Χ}^{2}+\mu_{Y}^{2}+C_{1}\right)\left(\sigma_{Χ}^{2}+\sigma_{Y}^{2}+C_{2}\right)}$$

where

- *μ_Χ_* and *μ_Y_* are the mean intensities of *X_output_* and *X_target_*,
- $\sigma_{Χ}^{2}\;\mathrm{and}\;\sigma_{Y}^{2}$ are the variances of *X_output_* and *X_target_*,
- *σ_XY_* is the covariance between the two images,
- *C*_1_ and *C*_2_ are small constants to avoid division by zero.

SSIM values range from 0 to 1, with higher values indicating better structural preservation [15,36,37].

Following these quantitative metrics by our physicists’ team, a qualitative evaluation by the enhancement of the scintigraphic image quality was reported by two nuclear medicine physicians (Observer 1 and Observer 2), with over five years of clinical experience in nuclear medicine. Both observers assessed the reconstructed images based on four criteria: noise level, visibility of key anatomical structures, structural detail preservation, and overall diagnostic confidence. Each criterion was rated on a scale from 1 to 5, with 1 representing the lowest quality and 5 representing the highest quality, as described in Table 1. To obtain an overall assessment of image quality, a total score was calculated by averaging the ratings across the four evaluation criteria. The final image quality score for each image was determined by computing the mean total score from both Observer 1 and Observer 2’s assessments. To assess the reliability of the qualitative evaluation, we calculated the intraclass correlation coefficient (ICC) between Observer 1 and Observer 2 across all dose levels. The analysis yielded an ICC(2,1) = 0.939, with a 95% confidence interval of (0.86–0.97) [38]. Additionally, a second quality assessment was conducted by Observer 3, a nuclear medicine physician with over five years of experience. Observer 3 conducted a Two-Alternative Forced Choice (2AFC) test [39]. In this test, Observer 3 was presented with a pair of images—one denoised and one original full-dose scan—and asked to select the preferred image based on overall perceived quality, without knowing or being informed about the actual origin. When the denoised image was preferred, the observer was asked to briefly state the reason for their choice. To reduce bias, the image order was randomized, and all identifying metadata were removed. A total of 147 image pairs per anatomical region (thorax and pelvis), spanning dose levels from 30% to 90%, were assessed.

This combination of quantitative (PSNR, SSIM) and qualitative (expert assessment) evaluations provides a comprehensive approach to assessing the model’s ability to generate both accurate and visually interpretable image reconstructions.

## 3. Results

To evaluate the performance of the algorithm, quantitative metrics (PSNR and SSIM) were used (Section 3.1) by nuclear medicine physicists, in cooperation with a qualitative assessment conducted by two nuclear medicine physicians with more than five years of professional experience (Section 3.2).

### 3.1. Quantitative Evaluation

**Figure 4:**
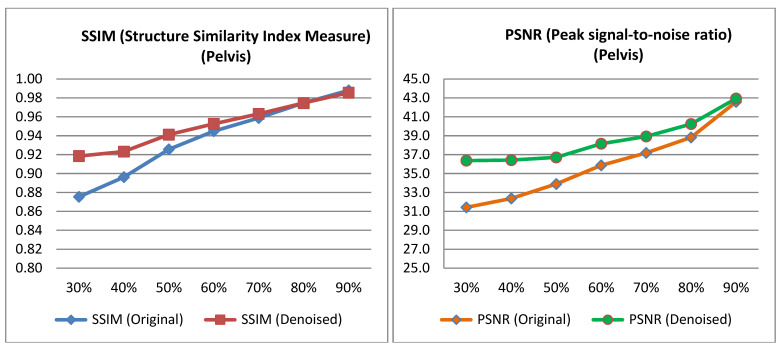
PSNR and SSIM variation across different low-dose percentages for pelvis images.

**Figure 5:**
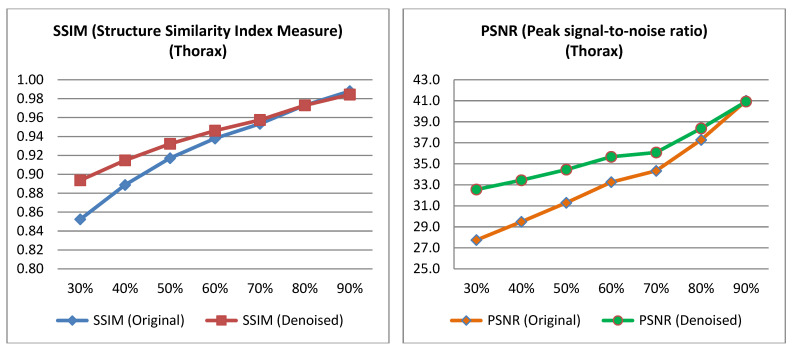
PSNR and SSIM variation across different low-dose percentages for thorax images.

The PSNR and SSIM indices were calculated for the pairs from the test set, as well as for the pairs derived from the low-dose images of the test set and their corresponding denoised images. The average value was then computed for each low-dose percentage from which the corresponding low-dose input image was derived for the algorithm. The Table 2 and Table 3 presents the mean PSNR and SSIM values, along with the *p*-value obtained from performing a *t*-test on the data [40]. Relevant Figure 4 and Figure 5 are also presented below.

### 3.2. Qualitative Evaluation

**Figure A1:**
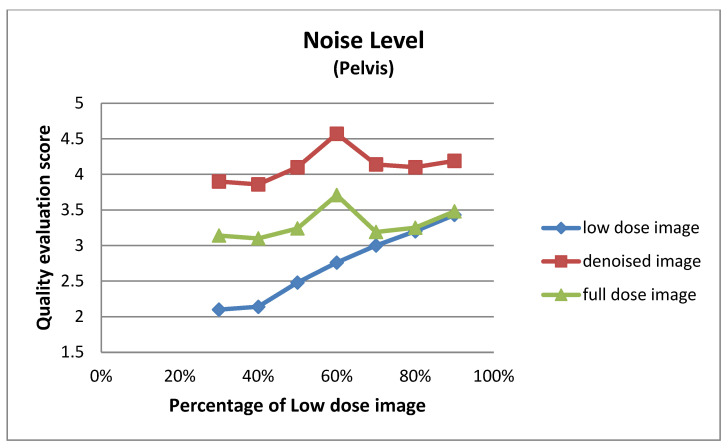
Noise level for pelvis images at different dose levels.

**Figure A2:**
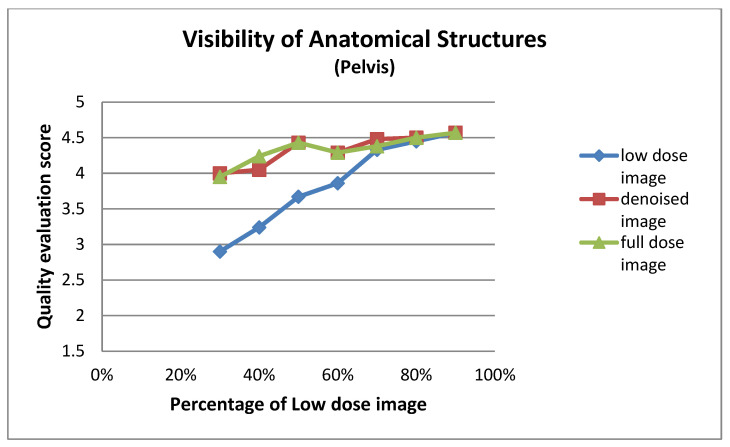
Visibility of anatomical structures for pelvis images at different dose levels.

**Figure A3:**
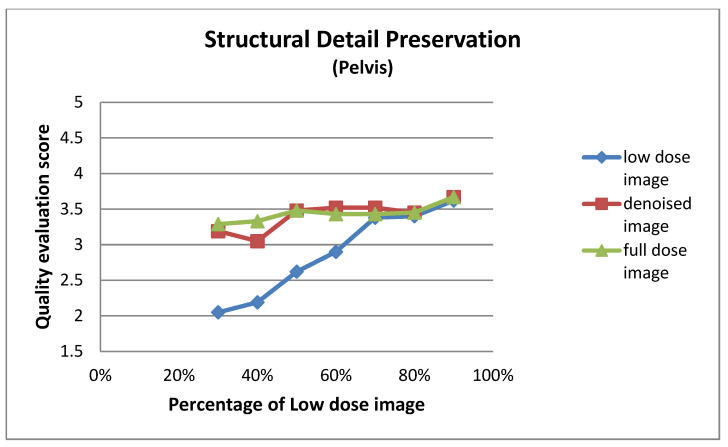
Structural detail preservation for pelvis images at different dose levels.

**Figure A4:**
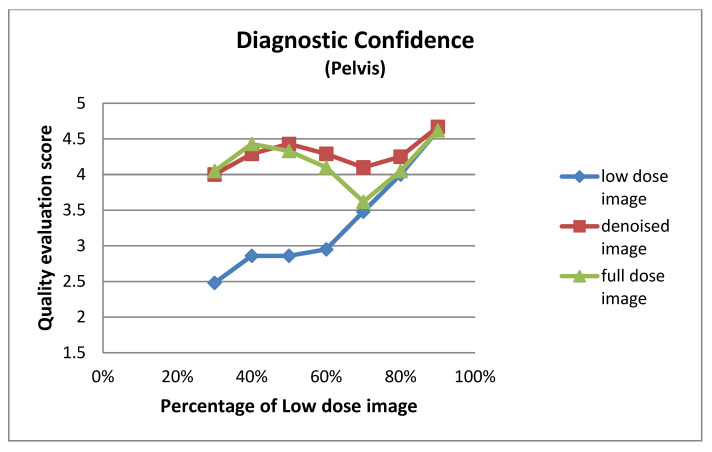
Diagnostic confidence for pelvis images at different dose levels.

**Figure A5:**
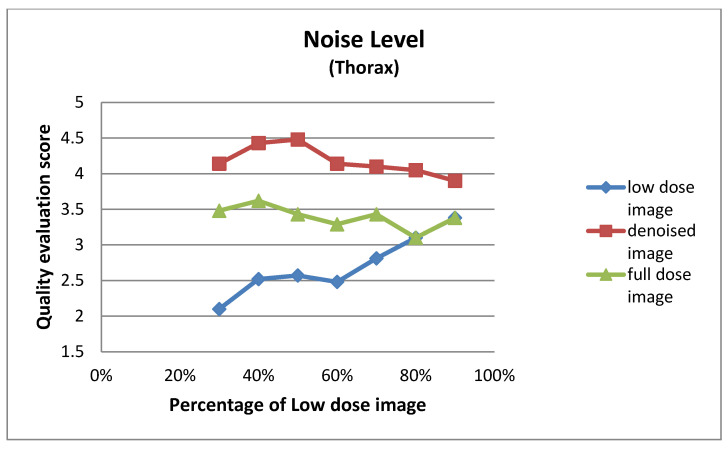
Noise level for thorax images at different dose levels.

**Figure A6:**
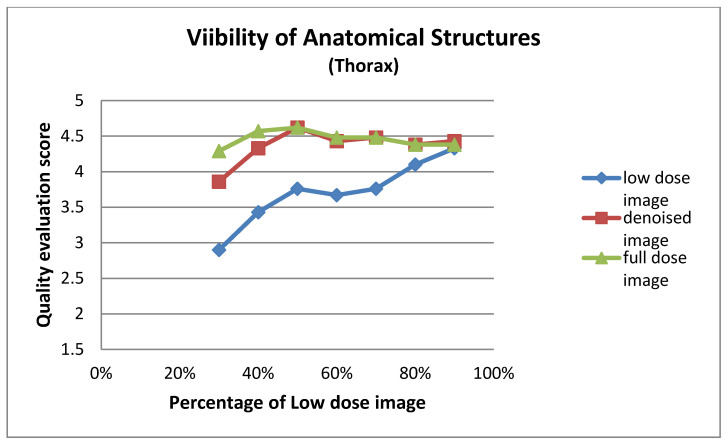
Visibility of anatomical structures for thorax images at different dose levels.

**Figure A7:**
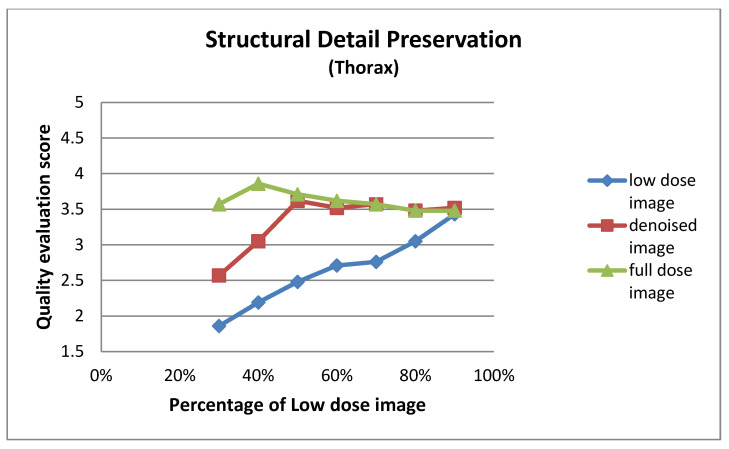
Structural detail preservation for thorax images at different dose levels.

**Figure A8:**
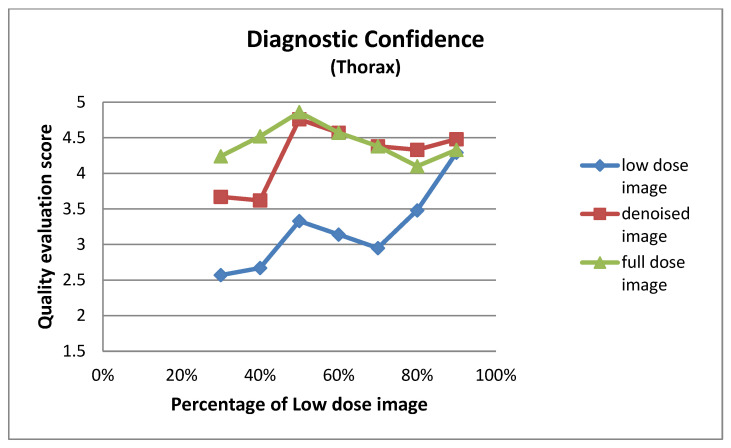
Diagnostic confidence for thorax images at different dose levels.

As described in the Section 2, two nuclear medicine physicians (Observer 1 and Observer 2), both with over five years of experience in nuclear medicine and the enhancement of scintigraphic image quality, evaluated the low-dose images, their corresponding ground truth images from the test set, and the denoised output images generated by the algorithm. They assessed four qualitative metrics: noise level, visibility of anatomical structures, preservation of image details, and overall diagnostic suitability, rating each criterion on a scale from 1 to 5. The overall quality score for each image was calculated by averaging the scores from the four metrics. The final image quality score for each image was determined by computing the mean total score from both experts’ evaluations. Additionally, a second evaluation was conducted by Observer 3, a nuclear medicine physician with over five years of experience. Observer 3 performed a Two-Alternative Forced Choice (2AFC) test, choosing between the denoised output and the original image based on preference, without knowledge of which image corresponded to each category. When Observer 3 selected the denoised output, reasons for the choice were provided. The results of these evaluations, including relevant tables and figures, are presented below, with the findings for each qualitative assessment metric across the two anatomical regions: pelvis and thorax. The mean scores for each of the four qualitative metrics, as assessed by both experts, are also presented in Appendix A (Table A2, Table A3, Table A4, Table A5, Table A6, Table A7, Table A8 and Table A9, Figure A1, Figure A2, Figure A3, Figure A4, Figure A5, Figure A6, Figure A7 and Figure A8), along with the corresponding tables and figures.

#### 3.2.1. Expert-Based Evaluation: Metrics for Image Quality Assessment

**Figure 6:**
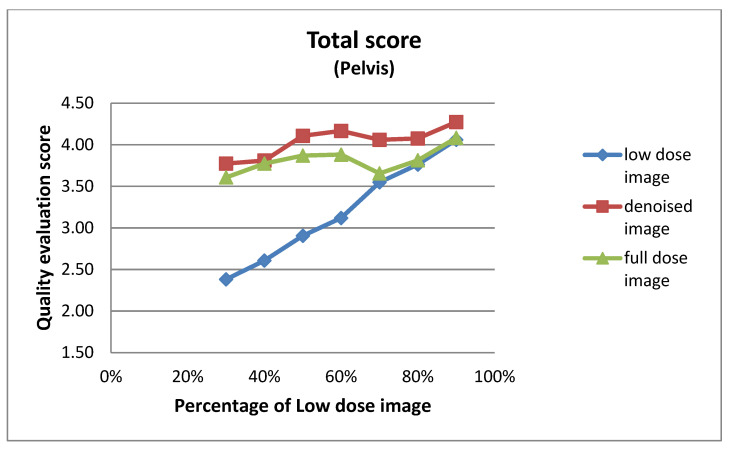
Total mean image quality scores across varying dose levels (30% to 90%) for pelvis images, as evaluated by two experts. Scores are averaged across four qualitative metrics: noise level, visibility of anatomical structures, preservation of image details, and overall diagnostic suitability. Three curves represent the low-dose images, denoised outputs, and full-dose reference images.

**Figure 7:**
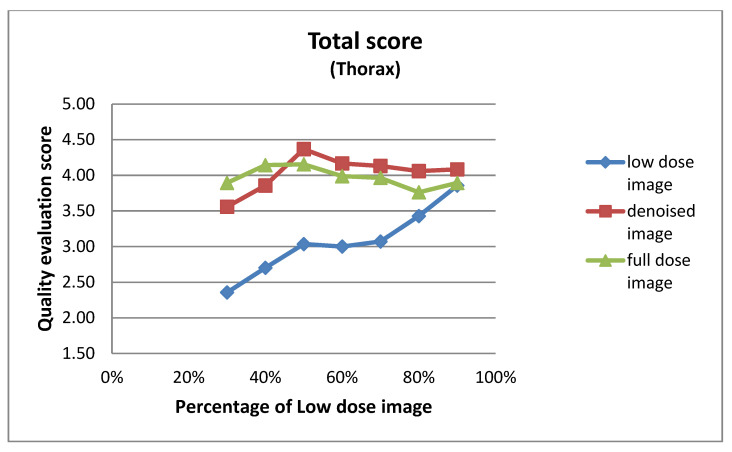
Total mean image quality scores across varying dose levels (30% to 90%) for thorax images, as evaluated by two experts. Scores are averaged across four qualitative metrics: noise level, visibility of anatomical structures, preservation of image details, and overall diagnostic suitability. Three curves represent the low-dose images, denoised outputs, and full-dose reference images.

**Figure 8:**
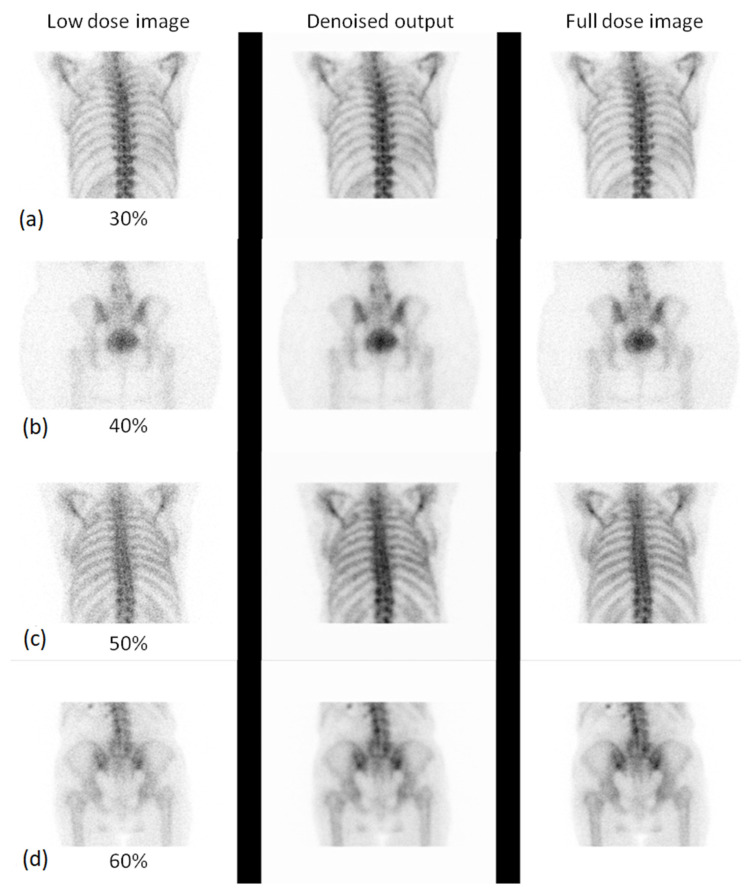
Visual comparison of low-dose, denoised, and full-dose images for different anatomical regions and dose levels. Each subpanel shows three images: the low-dose image, the denoised output, and the full-dose reference image. Specifically, subpanel (**a**) shows the thorax at the 30% low dose, subpanel (**b**) shows the pelvis at the 40% low dose, subpanel (**c**) shows the thorax at the 50% low dose, and subpanel (**d**) shows the pelvis at the 60% low dose.

This subsection details the expert-based assessment results, focusing on four qualitative metrics: noise level, visibility of anatomical structures, preservation of image details, and overall diagnostic suitability. As described earlier, two observers (Observer 1 and Observer 2) with extensive experience in nuclear medicine independently rated each image on a five-point scale. Figure 6 and Figure 7 present the total mean quality scores across the range of low-dose levels (30% to 90%) for thorax and pelvis regions, respectively. Each plot compares the low-dose images, denoised outputs, and corresponding full-dose references. The results show a consistent trend across both anatomical areas: the denoised images achieved higher quality scores than the original low-dose inputs, approaching the performance of the full-dose references, especially at mid-to-high dose levels. In the thorax region (Figure 7), the denoising algorithm showed substantial improvement over low-dose images, particularly between 30% and 50% dose levels. For example, at the 30% dose, the mean quality score increased from 2.36 (low-dose) to 3.56 (denoised), approaching the full-dose score of 3.89. While this improvement is substantial, it reflects perceived image quality rather than diagnostic accuracy, and should be interpreted cautiously. In the pelvis region (Figure 6), the improvement was even more pronounced; at the 30% dose, the score increased from 2.38 to 3.77, nearly matching or exceeding the full-dose reference score of 3.61. For instance, at the 90% dose, the denoised image was rated slightly higher than the corresponding full-dose image. This may be attributed to enhanced smoothness and noise suppression introduced by the denoising model, which was visually preferred by experts. However, this preference does not necessarily indicate superior diagnostic content and may reflect perceptual bias. In contrast, thoracic low-dose images exhibited more blurring and loss of fine detail, which may have limited the effectiveness of the denoising process in that region. To further illustrate the evaluation, Figure 8 presents representative image examples from both anatomical regions that were included in the expert assessment. While no individual comments are provided for these cases, each set includes the low-dose image, the corresponding denoised output, and the full-dose reference. Two cases from each region (thorax and pelvis) are shown, visually demonstrating how the denoising algorithm enhances image quality while preserving clinically relevant anatomical structures. Furthermore, to assess whether the denoising process preserved the perceived diagnostic confidence relative to the full-dose images, we analyzed expert ratings using both paired *t*-tests and the Two One-Sided Tests (TOST) procedure. These tests focused specifically on the overall diagnostic confidence metric. The paired *t*-test showed no statistically significant difference in diagnostic confidence between full-dose and denoised images in either anatomical region (thorax: *p* = 0.066; pelvis: *p* = 0.064), suggesting an equivalent perceptual diagnostic value. The TOST procedure, with equivalence bounds set at ±1 on the five-point Likert scale, confirmed statistical equivalence between the denoised and full-dose images in both regions (*p* < 0.001). These results support the interpretation that the model preserves the clinical utility of the exam, even at reduced dose levels [41].

#### 3.2.2. 2AFC Evaluation: Preference-Based Image Quality Assessment

**Figure 9:**
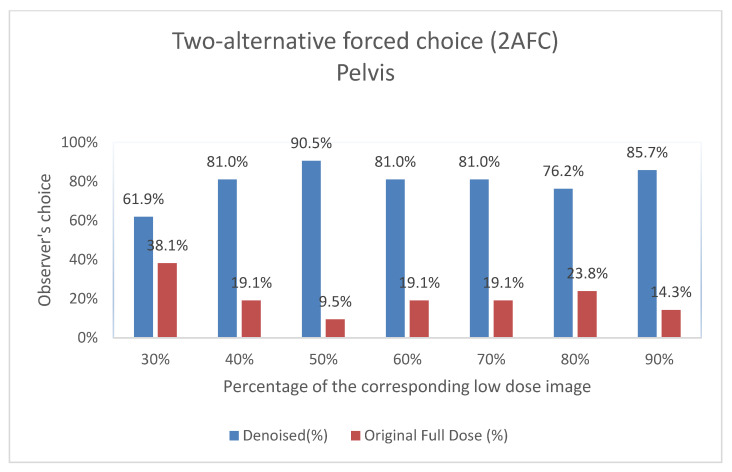
Two-Alternative Forced Choice (2AFC) Test for Pelvis Images. For each percentage of images evaluated, the graph shows the proportion of times the denoised output (denoted as “Denoised”) and the original image (denoted as “Original Full Dose”) were preferred by Observer 3. The data are shown as two columns representing the percentage of choices for each category.

**Figure 10:**
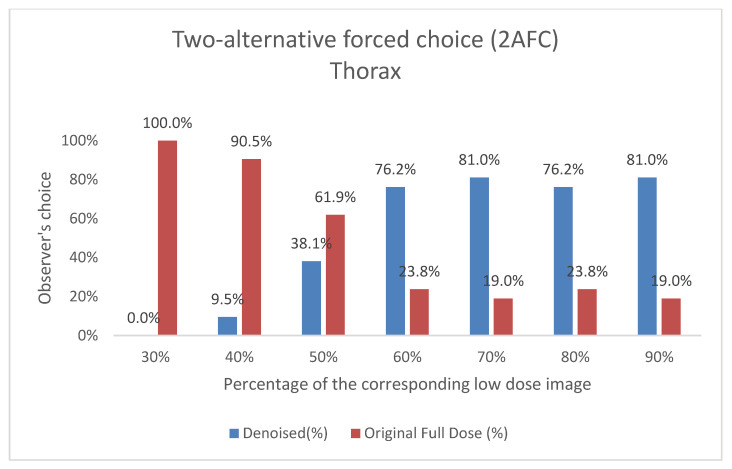
Two-Alternative Forced Choice (2AFC) Test for Thorax Images. For each percentage of images evaluated, the graph shows the proportion of times the denoised output (denoted as “Denoised”) and the original image (denoted as “Original Full Dose”) were preferred by Observer 3. The data are shown as two columns representing the percentage of choices for each category.

This subsection presents the results of the Two-Alternative Forced Choice (2AFC) evaluation conducted by Observer 3. In this blind test, Observer 3 was asked to choose between the denoised output and the original full-dose image based on overall preference, without knowledge of which image corresponded to each category. Figure 9 and Figure 10 show the results of this preference-based assessment, indicating the percentage of times the denoised output and the original low-dose image were selected across different dose levels for both the thorax and pelvis regions. The results demonstrate a clear preference for the denoised images, particularly as the low-dose percentage increases.

**Figure 11:**
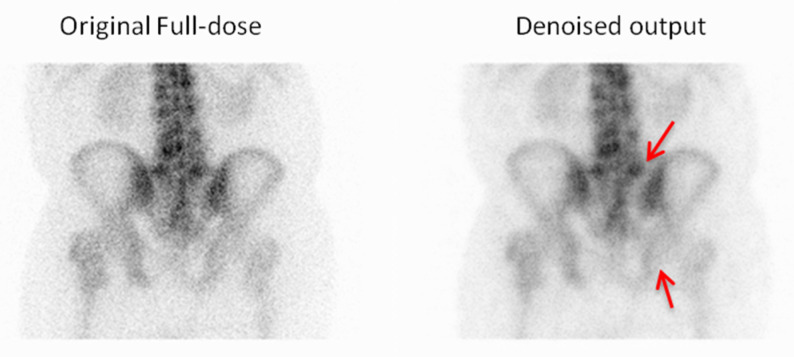
For the first example, the observer chose the denoised image due to its improved visibility of the vertebral endplates and a clearer depiction of the sacroiliac joints, which enhanced the anatomical assessment. Red arrows indicate the vertebral endplates and sacroiliac joints.

**Figure 12:**
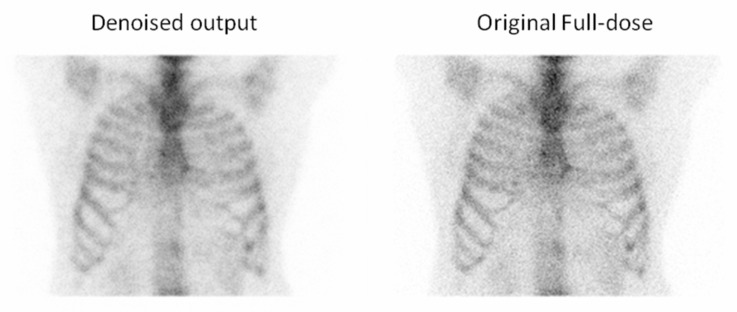
For the second example, the observer selected the denoised image due to the reduced noise compared to the full-dose image, which enhanced the overall image quality and clarity.

**Figure 13:**
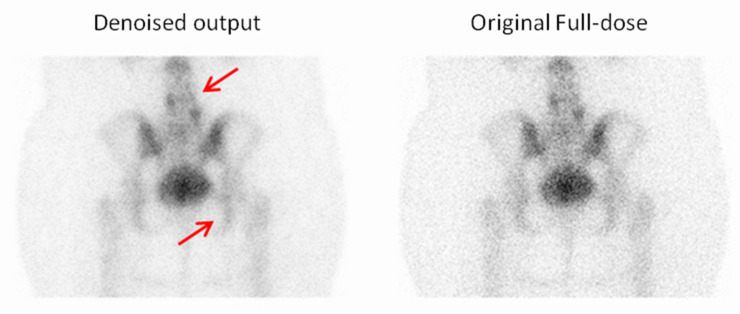
For the third example, the observer selected the denoised image due to the reduced noise compared to the full-dose image, which enhanced the overall image quality and clarity. Red arrows indicate two focal regions in the image that demonstrate noticeable smoothing resulting from noise reduction.

**Figure 14:**
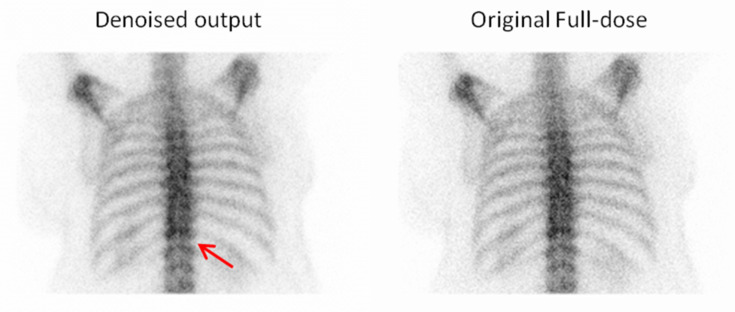
For the fourth example, the observer selected the denoised image due to its superior visibility of the intervertebral discs, which facilitated better anatomical recognition and assessment. Red arrow indicate an intervertebral disc.

In addition to the preference selections, Observer 3 also provided comments on specific cases where the denoised output was favored over the original full-dose image (Figure 11, Figure 12, Figure 13 and Figure 14). These remarks were given without knowledge of the image category and offer qualitative insight into the reasons behind the choices, further supporting the perceived diagnostic benefits of the denoised images.

## 4. Discussion

### 4.1. Summary of Main Findings

This study demonstrated that the proposed Convolutional Autoencoder—enhanced with multiscale feature blocks, channel attention, and efficient residual connections—effectively improves image quality in low-dose planar bone scintigraphy acquired under real clinical acquisition constraints. Across thorax and pelvis datasets, the model yielded significant improvements in SSIM and PSNR, particularly at 30–70% dose levels, demonstrating strong capacity to recover diagnostic details from highly noisy input images (Table 2 and Table 3). Visual evaluation, including 2AFC testing and total score assessments by nuclear medicine physicians, revealed a consistent preference for denoised images over original full-dose images. In some cases, denoised outputs were subjectively rated as superior to full-dose scans, underscoring the potential for deep learning to enhance diagnostic confidence beyond simple noise suppression.

### 4.2. Comparison with Previous Studies

Our findings show that deep learning-based denoising significantly enhances image quality in bone scintigraphy images acquired with shortened acquisition times to simulate low-dose conditions. This is consistent with recent work in the field of nuclear medicine image enhancement using AI, yet distinct in its clinical realism and methodological breadth. Compared to prior studies focused on bone scintigraphy, our use of real low-dose data (from shortened acquisition rather than synthetic downsampling) adds practical relevance. For instance, Murata et al. (2024) [7] applied deep learning to enhance artificially degraded bone scans, showing quality gains under simulated conditions. In contrast, our use of real patient data under reduced acquisition reflects clinical workflows more accurately and highlights the model’s robustness in diverse scenarios, including the presence of pathologies and medical devices—factors often excluded in synthetic datasets. Similarly, Ito et al. (2022) [6] employed list-mode data to mimic dose reduction, achieving high fidelity in image reconstructions using super-resolution CNNs. However, their evaluation relied exclusively on quantitative metrics (e.g., SSIM, PSNR), whereas our study integrates both quantitative and qualitative analyses, including multi-observer scoring and 2AFC preference testing, providing a more clinically grounded validation. Further supporting our findings, Kovács et al. (2022) [42] highlighted the robustness of deep learning-based denoising across a wide range of noise levels in bone scintigraphy, and demonstrated that such approaches outperform conventional filters, even in the absence of noise-free reference images. Csikos et al. (2024) [43] also showed that an AI-based noise-reduction filter could significantly improve image quality in low-count whole-body bone scintigraphy. However, their evaluation relied entirely on qualitative metrics—subjective scoring by nuclear medicine physicians—without quantitative assessment of fidelity or noise suppression. Our study advances both of these efforts by integrating a broad range of real-world clinical scenarios and combining subjective and objective evaluation criteria, thereby offering a more holistic and reproducible measure of clinical utility. By leveraging a dataset that spans a wide spectrum of clinical presentations, we further ensure generalizability and reduce the risk of model bias. Moreover, Minarik et al. (2019) [44] used Monte Carlo simulations to denoise bone scintigraphy images but excluded real-world complexities, limiting their model’s direct clinical applicability. We address this gap by validating on actual clinical data with varied anatomical and pathological presentations.

Beyond skeletal imaging, our work aligns with studies in other scintigraphy domains. For example, Arsénio et al. (2025) [45] showed that deep learning can successfully recover image quality in pediatric renal scintigraphy under low-dose conditions. Similarly, Ichikawa et al. (2023) [46] applied AI to reduce acquisition times in pediatric ^99m^Tc-DMSA scans, achieving substantial time savings without degrading diagnostic confidence. In myocardial perfusion SPECT, Ramon et al. (2020) [8] used convolutional denoising networks to maintain diagnostic accuracy even with reduced dose protocols. These studies reinforce the versatility of AI in nuclear medicine and validate its potential to optimize acquisition protocols across imaging modalities and patient populations. 

Interestingly, while our study focuses on planar bone scintigraphy and utilizes an autoencoder-like architecture, the quantitative improvements we observed—particularly in SSIM at low-dose levels (e.g., pelvis SSIM from 0.875 to 0.918 at 30% dose) (Table 2 and Table 3), are methodologically comparable to outcomes reported in PET denoising studies. For instance, Li et al. (2021) [13] and Ahmad et al. (2023) [47] demonstrated that GAN-based approaches can enhance image quality in PET by recovering fine structural details in low-dose conditions. Similarly, Gong et al. (2017) [48] introduced an early CNN-based denoising model for PET trained on full-dose/low-dose pairs, an approach echoed in our supervised learning strategy. Xu et al. (2017) [49] used deep residual networks to reconstruct PET images from ultra-low-dose inputs, underscoring the general potential of deep learning in extreme noise conditions. Hybrid approaches also show promise: Kim et al. (2018) [50] incorporated deep priors into PET image reconstruction, blending traditional and AI-driven techniques, while Katsari et al. (2021) [51] demonstrated successful deployment of AI-enhanced PET imaging in clinical workflows. In terms of clinical validation, Bonardel et al. (2022) [11] and Weyts et al. (2022) [52] both validated AI-based PET denoising algorithms in clinical and phantom settings, demonstrating significant improvements that allowed dose or time reductions without compromising diagnostic interpretability. While PET and planar scintigraphy differ in data characteristics and clinical usage, these studies highlight important methodological similarities, reinforcing the relevance and generalizability of deep learning-based denoising across nuclear medicine modalities. Our own observer-based evaluations show similar trends; for example, in pelvis scans at a 50% dose, over 90% of responses favored the denoised images compared to the original full-dose ones (Figure 9), suggesting not only recovery but potential enhancement of diagnostic quality. Collectively, these results support the general effectiveness and adaptability of deep learning-based denoising within nuclear medicine. Our study adds to this growing body of work by presenting a clinically validated, robust, and interpretable framework for dose and time reduction in planar bone scintigraphy. The model’s strong performance—achieved without adversarial loss or complex generative designs—emphasizes the importance of well-structured training datasets and comprehensive evaluation strategies in producing clinically meaningful outcomes.

### 4.3. Clinical Interpretation and Reader Evaluation

The results of the expert visual evaluations further support the effectiveness of the proposed model. In the 2AFC tests, denoised images were preferred over full-dose images in the majority of comparisons, indicating favorable perceptual quality. In some scenarios, denoised outputs not only matched but exceeded the visual preference scores of full-dose images (Figure 9 and Figure 10). For example,

- Pelvis at a 50% dose: 90.48% of comparisons favored the denoised image.
- Thorax at a 70% dose: 81% of comparisons favored the denoised image.

These findings suggest that the denoising process preserved—and, as seen in the examples above, in some cases enhanced—visual features considered diagnostically important by nuclear medicine experts. However, as noted earlier, these results reflect subjective preferences and do not imply improved diagnostic accuracy. Notably, this trend was most prominent at intermediate dose levels (50–70%), where denoised images often approximated or even exceeded the perceptual quality of full-dose scans. This effect may be attributed to the combined impact of noise suppression and smoothing, where the denoising network reduces acquisition-related artifacts that can persist even in full-dose images. A similar pattern was observed in the total score evaluations: scores consistently increased from low-dose to denoised to full-dose images, demonstrating the model’s ability to restore image quality toward baseline. Interestingly, in the pelvis region at the 90% dose, the denoised image scored slightly higher than the full-dose image (4.27 vs. 4.08), further reinforcing the subjective appeal of the enhanced outputs (Figure 6 and Figure 7). As described in Section 3.2.1, both paired *t*-tests and equivalence testing (TOST) confirmed that the diagnostic confidence associated with denoised images was statistically equivalent to that of full-dose images. These findings support the conclusion that the denoising model maintains the clinical utility of the examination—an essential criterion for any dose-reduction strategy in nuclear medicine imaging.

### 4.4. Strengths and Contributions

This study presents several key strengths and contributions to the field. Firstly, the use of real clinical data, rather than simulated noise, increases the practical relevance of the findings, ensuring that they are directly applicable to real-world clinical settings. Additionally, the novel network design, combining multiscale features, attention mechanisms, and residual connections, demonstrated strong performance across various anatomical regions, highlighting the model’s versatility and robustness. The evaluation process was comprehensive, employing both objective metrics (SSIM and PSNR) and subjective visual reader assessments, ensuring a thorough validation of the model’s effectiveness. Furthermore, the study shows that AI-based enhancement can match or even surpass the quality of full-dose images across multiple anatomical regions, especially at intermediate dose levels.

### 4.5. Limitations

While the results of this study are promising, several limitations should be acknowledged. Although the dataset included images acquired from two different gamma cameras—the Siemens Symbia E Dual Head System (Siemens Healthineers, Erlangen, Germany, 2014) and a Siemens E-CAM e-signature (Siemens Healthineers, Erlangen, Germany, 2008)—all data were collected in 2023 at a single clinical center (Nuclear Medicine Department, University General Hospital of Alexandroupolis), which may limit the generalizability of the model across institutions, scanner vendors, and broader patient populations. Future work should incorporate multi-center datasets to validate the robustness of the model in diverse clinical environments. Additionally, this evaluation focused primarily on general visual quality. While the current study does not include objective diagnostic performance metrics such as sensitivity or specificity for lesion detection, it uses a validated clinical proxy—the overall diagnostic confidence score—rated by nuclear medicine physicians. This metric reflects perceived ease of lesion identification and characterization and has been statistically shown to be equivalent between denoised and full-dose images. Therefore, the study provides meaningful and sufficient evidence for assessing the clinical utility of the algorithm in its current form. While the study utilized a reasonably sized dataset, a larger dataset incorporating a broader variety of anatomical areas and pathologies could provide more robust results. This would also ensure that the model generalizes well to different patient conditions and anatomical regions. An important consideration for future work is whether it would be more effective to develop a single general model that works across all anatomical areas or separate models for different anatomical regions. While a general model may be more efficient and easier to deploy, separate models could potentially provide more optimized performance for specific anatomical regions, ensuring the highest quality for each case.

### 4.6. Clinical Implications and Future Directions

The findings of this study have significant clinical implications and open avenues for future research. Integrating deep learning-based denoising into nuclear medicine workflows could reduce radiation exposure by enabling diagnostic-quality imaging at lower radiopharmaceutical doses, helping to minimize radiation exposure not only for patients but also for clinical staff and accompanying family members. This approach is particularly relevant in routine clinical practice, where cumulative exposure is a concern. Additionally, shorter acquisition times can enhance patient comfort by reducing the need for prolonged stillness, which also lowers the risk of motion artifacts. Beyond these benefits, this approach may also improve workflow efficiency in high-volume centers by shortening scan durations and increasing patient throughput. However, these operational benefits and their economic implications require validation through prospective clinical implementation studies. Future research should focus on further optimizing network generalization and integration into clinical workflows. While the current study does not explicitly evaluate lesion detection performance, it demonstrates that the denoised images maintain the diagnostic confidence required for clinical decision making, thus introducing a crucial step toward safe dose reduction in nuclear medicine. Multi-center studies across different institutions and imaging systems are also essential to assess generalizability and robustness. Expanding the approach to other nuclear medicine modalities (e.g., PET or tomographic-dynamic SPECT) and exploring real-world clinical deployment strategies will be critical for translating these findings into clinical practice.

## 5. Conclusions

In this study, we investigated the potential of deep learning-based denoising to enhance the quality of low-dose nuclear medicine images. Using real clinical data, our Enhanced Convolutional Autoencoder (ECAE) demonstrated substantial improvements in image quality, as measured by both quantitative metrics (PSNR and SSIM) and expert-based qualitative evaluations. Unlike previous studies that relied on synthetic noise, our approach employed real low-dose acquisitions, offering greater clinical realism and applicability. The results indicate that the proposed model can effectively preserve anatomical detail and reduce noise, potentially allowing diagnostic-quality imaging at reduced dose levels. This suggests a path forward for minimizing patient radiation exposure without compromising visual interpretability. Future studies should incorporate multi-reader assessments and expand the evaluation to multi-institutional datasets.

## Figures and Tables

**Table 1:** Qualitative evaluation criteria and scoring scale (1 = lowest quality, 5 = highest quality).

Criteria	1	2	3	4	5
Noise Level	Very high—severe noise, difficult to interpret	High—noticeable noise	Moderate—acceptable noise	Low—minimal noise	Very low—almost no noise
Visibility of Key Anatomical Structures	Not visible at all	Poorly visible	Moderately visible	Clearly visible	Perfectly visible
Structural Detail Preservation	Severe loss of detail	Moderate loss	Acceptable loss	Well-preserved	Fully preserved
Overall Diagnostic Confidence	Not confident at all	Low confidence	Neutral	Confident	Very confident

**Table 2:** SSIM and PSNR metrics for denoised and original pelvis images across different low-dose percentages with corresponding *p*-values.

Pelvis						
% Low Dose Image	SSIM (Original)	SSIM (Denoised)	*p*-Value	PSNR (Original)	PSNR (Denoised)	*p*-Value
30%	0.875	0.918	<0.005	31.43	36.37	<0.005
40%	0.896	0.923	<0.005	32.37	36.42	<0.005
50%	0.926	0.941	<0.005	33.90	36.71	<0.005
60%	0.945	0.953	<0.005	35.87	38.15	<0.005
70%	0.959	0.963	<0.005	37.19	38.92	<0.005
80%	0.975	0.975	0.977	38.82	40.24	<0.005
90%	0.988	0.988	0.985	42.58	42.94	0.192

**Table 3:** SSIM and PSNR metrics for denoised and original thorax images across different low-dose percentages with corresponding *p*-values.

Thorax						
% Low Dose Image	SSIM (Original)	SSIM (Denoised)	*p*-Value	PSNR (Original)	PSNR (Denoised)	*p*-Value
30%	0.852	0.894	<0.005	27.75	32.56	<0.005
40%	0.889	0.915	<0.005	29.48	33.44	<0.005
50%	0.917	0.932	<0.005	31.29	34.45	<0.005
60%	0.938	0.946	<0.005	33.25	35.67	<0.005
70%	0.953	0.957	<0.005	34.33	36.08	<0.005
80%	0.973	0.973	0.977	37.28	38.37	<0.005
90%	0.988	0.988	0.982	41.01	40.93	0.769

## Data Availability

The data that support the findings of this study are available from the corresponding author upon reasonable request.

## Appendix A

**Table A1:** Comparison of SSIM and PSNR between scanner vendors and anatomical regions.

Metric	Region	E-Cam	Symbia
SSIM	Pelvis	0.944	0.934
SSIM	Thorax	0.933	0.928
PSNR	Pelvis	36.17	35.99
PSNR	Thorax	33.63	33.38

**Table A2:** Noise level scores from qualitative evaluation of pelvis images across dose levels.

Low Dose Image Percentage	Noise Level (Pelvis)		
	Low-Dose	Denoised	Full-Dose
30%	2.10 ± 0.54	3.90 ± 0.54	3.14 ± 0.48
40%	2.14 ± 0.48	3.86 ± 0.36	3.10 ± 0.54
50%	2.48 ± 0.51	4.10 ± 0.30	3.24 ± 0.44
60%	2.76 ± 0.54	4.57 ± 0.51	3.71 ± 0.56
70%	3.00 ± 0.32	4.14 ± 0.36	3.19 ± 0.51
80%	3.20 ± 0.41	4.10 ± 0.31	3.25 ± 0.44
90%	3.43 ± 0.51	4.19 ± 0.40	3.48 ± 0.51

**Table A3:** Visibility of anatomical structures scores from qualitative evaluation of pelvis images across dose levels.

Low Dose Image Percentage	Visibility of Anatomical Structures (Pelvis)		
	Low-Dose	Denoised	Full-Dose
30%	2.90 ± 0.94	4.00 ± 0.84	3.95 ± 0.86
40%	3.24 ± 0.70	4.05 ± 0.80	4.24 ± 0.77
50%	3.67 ± 0.73	4.43 ± 0.51	4.43 ± 0.51
60%	3.86 ± 0.65	4.29 ± 0.46	4.29 ± 0.46
70%	4.33 ± 0.48	4.48 ± 0.51	4.38 ± 0.50
80%	4.45 ± 0.51	4.50 ± 0.51	4.50 ± 0.51
90%	4.57 ± 0.51	4.57 ± 0.51	4.57 ± 0.51

**Table A4:** Structural detail preservation scores from qualitative evaluation of pelvis images across dose levels.

Low Dose Image Percentage	Structural Detail Preservation (Pelvis)		
	Low-Dose	Denoised	Full-Dose
30%	2.05 ± 0.92	3.19 ± 1.03	3.29 ± 1.01
40%	2.19 ± 0.75	3.05 ± 0.92	3.33 ± 0.80
50%	2.62 ± 0.74	3.48 ± 0.51	3.48 ± 0.51
60%	2.90 ± 0.70	3.52 ± 0.68	3.43 ± 0.68
70%	3.38 ± 0.50	3.52 ± 0.60	3.43 ± 0.60
80%	3.40 ± 0.50	3.45 ± 0.60	3.45 ± 0.60
90%	3.62 ± 0.59	3.67 ± 0.58	3.67 ± 0.58

**Table A5:** Diagnostic confidence scores from qualitative evaluation of pelvis images across dose levels.

Low Dose Image Percentage	Diagnostic Confidence (Pelvis)		
	Low-Dose	Denoised	Full-Dose
30%	2.48 ± 1.08	4.00 ± 0.95	4.05 ± 0.92
40%	2.86 ± 0.73	4.29 ± 0.64	4.43 ± 0.60
50%	2.86 ± 0.91	4.43 ± 0.75	4.33 ± 0.80
60%	2.95 ± 0.67	4.29 ± 0.64	4.10 ± 0.62
70%	3.48 ± 0.75	4.10 ± 0.70	3.62 ± 0.67
80%	4.00 ± 0.79	4.25 ± 0.64	4.05 ± 0.76
90%	4.62 ± 0.50	4.67 ± 0.48	4.62 ± 0.50

Subjective assessment of image quality based on four criteria: noise level, visibility of anatomical structures, preservation of structural details, and diagnostic confidence. Scores represent the average ratings from expert readers. The total score is calculated as the mean of these four metrics.

**Table A6:** Noise level scores from qualitative evaluation of thorax images across dose levels.

Low Dose Image Percentage	Noise Level (Thorax)		
	Low-Dose	Denoised	Full-Dose
30%	2.10 ± 0.62	4.14 ± 0.48	3.48 ± 0.51
40%	2.52 ± 0.51	4.43 ± 0.51	3.62 ± 0.50
50%	2.57 ± 0.60	4.48 ± 0.51	3.43 ± 0.51
60%	2.48 ± 0.60	4.14 ± 0.48	3.29 ± 0.46
70%	2.81 ± 0.40	4.10 ± 0.44	3.43 ± 0.51
80%	3.10 ± 0.30	4.05 ± 0.22	3.10 ± 0.30
90%	3.38 ± 0.67	3.90 ± 0.54	3.38 ± 0.67

**Table A7:** Visibility of anatomical structure scores from qualitative evaluation of thorax images across dose levels.

Low Dose Image Percentage	Visibility of Anatomical Structures (Thorax)		
	Low-Dose	Denoised	Full-Dose
30%	2.90 ± 0.94	3.86 ± 0.73	4.29 ± 0.56
40%	3.43 ± 0.75	4.33 ± 0.58	4.57 ± 0.60
50%	3.76 ± 0.70	4.62 ± 0.50	4.62 ± 0.50
60%	3.67 ± 0.80	4.43 ± 0.60	4.48 ± 0.60
70%	3.76 ± 0.70	4.48 ± 0.68	4.48 ± 0.68
80%	4.10 ± 0.54	4.38 ± 0.50	4.38 ± 0.50
90%	4.33 ± 0.66	4.43 ± 0.51	4.38 ± 0.59

**Table A8:** Structural detail preservation scores from qualitative evaluation of thorax images across dose levels.

Low Dose Image Percentage	Structural Detail Preservation (Thorax)		
	Low-Dose	Denoised	Full-Dose
30%	1.86 ± 0.73	2.57 ± 0.75	3.57 ± 0.75
40%	2.19 ± 0.75	3.05 ± 0.50	3.86 ± 0.65
50%	2.48 ± 0.60	3.62 ± 0.74	3.71 ± 0.78
60%	2.71 ± 0.78	3.52 ± 0.75	3.62 ± 0.74
70%	2.76 ± 0.70	3.57 ± 0.81	3.57 ± 0.81
80%	3.05 ± 0.59	3.48 ± 0.68	3.48 ± 0.60
90%	3.43 ± 0.68	3.52 ± 0.51	3.48 ± 0.60

**Table A9:** Diagnostic confidence scores from qualitative evaluation of thorax images across dose levels.

Low Dose Image Percentage	Diagnostic Confidence (Thorax)		
	Low-Dose	Denoised	Full-Dose
30%	2.57 ± 0.81	3.67 ± 0.73	4.24 ± 0.62
40%	2.67 ± 0.58	3.62 ± 0.59	4.52 ± 0.60
50%	3.33 ± 0.66	4.76 ± 0.44	4.86 ± 0.36
60%	3.14 ± 0.79	4.57 ± 0.60	4.57 ± 0.68
70%	2.95 ± 0.80	4.38 ± 0.74	4.38 ± 0.74
80%	3.48 ± 0.81	4.33 ± 0.66	4.10 ± 0.77
90%	4.29 ± 0.85	4.48 ± 0.60	4.33 ± 0.73

Subjective assessment of image quality based on four criteria: noise level, visibility of anatomical structures, preservation of structural details, and diagnostic confidence. Scores represent the average ratings from expert readers. The total score is calculated as the mean of these four metrics.
